# Minimal-invasive Stabilisation von Azetabulumfrakturen mit virtueller Navigation in Kombination mit robotergestützter 3-D-Bildgebung

**DOI:** 10.1007/s00064-024-00872-8

**Published:** 2024-11-11

**Authors:** Raffael Cintean, K. Schütze, F. Gebhard, C. Pankratz

**Affiliations:** Abteilung für Unfall‑, Hand‑, Plastische und Wiederherstellungschirurgie, Universitätsklinikum Ulm, Albert-Einstein-Allee 23, 89081 Ulm, Deutschland

**Keywords:** Azetabulumfraktur, Minimal-invasiv, 3‑D-Bildgebung, Navigation, Roboter, Roboterassistiert, Acetabular fracture, Minimally invasive surgical procedures, Three-dimensional imaging, Robots, Robot-assisted

## Abstract

**Operationsziel:**

Die minimal-invasive Stabilisierung von nicht und minimal dislozierten Azetabulumfrakturen unter Anwendung intraoperativer, robotergestützter 3‑D-Bildgebung sowie eines Navigationssystems.

**Indikationen:**

Nicht oder nur minimal dislozierte Frakturen des Azetabulums.

**Kontraindikationen:**

Trümmer- und grob dislozierte Frakturen des Azetabulums, Protrusion des Hüftkopfes in das Becken mit der Notwendigkeit der offenen Reposition, fehlende Möglichkeit der intraoperativen Navigation.

**Operationstechnik:**

Nach Lagerung des Patienten wird in die Spina iliaca anterior superior die patientenseitige Navigationsreferenz durch eine Schanz-Schraube befestigt. Anschließend können der 3‑D-Scan sowie die Registrierung des Datensatzes im Navigationssystem durchgeführt werden. Damit können die 7,3-mm-Schrauben über die 3‑D-Bildgebung geplant und anschließend durch Stichinzisionen implantiert werden.

**Weiterbehandlung:**

Bei erfolgreicher Schraubenimplantation kann der Patient durch die minimal-invasive Operationstechnik am Folgetag schmerzadaptiert unter physiotherapeutischer Beübung mobilisiert werden. In der Regel ist eine Vollbelastung möglich.

**Ergebnisse:**

Zwischen 2015 und 2023 wurden 101 Patienten mittels minimal-invasiver und navigationsgestützter Schraubenosteosynthese bei Azetabulumfrakturen versorgt. Bei 2 Patienten kam es nach Mobilisation zu einer sekundären Schraubendislokation in das Hüftgelenk, weshalb eine Revisionsoperation mit Neuplatzierung der Schraubenosteosynthese bzw. die Indikation zur Hüft-TEP notwendig war. Die minimal-invasive navigierte Schraubenosteosynthese bietet somit bei korrekter Indikationsstellung sowie Technik eine adäquate Möglichkeit der Versorgung von un- und minimal dislozierten Azetabulumfrakturen.

## Abstract (en)

**Objective:**

Minimally invasive stabilization of non- and minimally displaced acetabular fractures using intraoperative, robot-assisted three-dimensional (3D) imaging and a navigation system.

**Indications:**

Nondisplaced or only minimally displaced fractures of the acetabulum.

**Contraindications:**

Comminuted and highly displaced fractures of the acetabulum, protrusion of the femoral head into the pelvis with the need for open reduction, lack of possibility of intraoperative navigation.

**Surgical technique:**

After supine positioning the patient, the patient-side navigation reference is attached to the anterior superior iliac spine using a Schanz screw. The 3D scan and registration of the dataset in the navigation system can then be performed. This allows the 7.3 mm screws to be planned using 3D imaging and then implanted through minimally invasive incisions.

**Postoperative management:**

After successfully implanting the screws using the minimally invasive surgical technique, the patient can be mobilized the following day with pain-adapted physiotherapy exercises. Full weight bearing is usually possible.

**Results:**

Between 2015 and 2023, 101 patients were treated using minimally invasive and navigation-assisted screw osteosynthesis for acetabular fractures. In 2 patients, a secondary screw dislocation occurred in the hip joint after mobilization, which required revision surgery with repositioning of the screw osteosynthesis and a hip arthroplasty, respectively. Minimally invasive navigated screw osteosynthesis, thus, offers adequate treatment of nondisplaced and minimally displaced acetabular fractures. Attention must be paid to the correct indication and surgical technique.

## Vorbemerkungen

Azetabulumfrakturen sind komplexe Verletzungen, deren Behandlung die Operateure häufig vor Herausforderungen stellt. Mit dem demografischen Wandel zeigt sich auch die Häufigkeit der komplexen Frakturen bis ins hohe Alter in steigender Anzahl [2]. Hinzu kommt, dass diese Patienten häufig wegen chronischer Begleiterkrankungen behandelt werden, was das perioperative Risiko erhöht [5]. Das herkömmliche chirurgische Verfahren über die offene Reposition und die anschließende Plattenosteosynthese können gerade bei älteren Patienten aufgrund der Größe der Operation mit erheblicher Morbidität und entsprechenden Komplikationen verbunden sein [3]. Die minimal-invasive Schraubenosteosynthese bietet eine vielversprechende Alternative, die eine stabile Fixierung ermöglicht und gleichzeitig das Weichteiltrauma minimiert und das Risiko intra- und postoperativer Komplikationen verringert [4]. Dies bietet zwar insbesondere für ältere Patienten durch die geringere intraoperative Belastung einen Vorteil, kann jedoch auch bei jüngeren Patienten angewandt werden [7]. Bei dieser Technik werden die Schrauben mittels Navigationssystemen intraoperativ geplant. Anschließend werden die Schrauben über navigiert implantierte Führungsdrähte die Fraktur fixieren. Ein vom Operateur autark bedientes robotergestütztes bildgebendes Verfahren ermöglicht zudem die hochauflösende und exakte Durchleuchtung sowie die Durchführung eines 3‑D-Scans. Somit kann durch automatisches Anfahren des Röntgengerätes an gespeicherte Positionen nicht nur die Strahlenbelastung für OP-Personal sowie Patient reduziert werden, auch verkürzen sich die Operationszeit und somit die intraoperative Belastung für die Patienten auf ein Minimum.

## Operationsprinzip und -ziel

Die robotergestützte und navigierte und Versorgung von nicht und minimal dislozierten Azetabulumfrakturen bietet eine minimal-invasive Möglichkeit der Versorgung von einfachen und mehrfragmentären Azetabulumfrakturen.

## Vorteile

- Die minimal-invasive Versorgung von nicht und minimal dislozierten Azetabulumfrakturen bietet eine schonende Alternative der operativen Stabilisierung, welche sonst meist über einen komplexen Zugang mit großem Morbiditätsrisiko zum Becken durchgeführt wird.
- Durch die minimal-invasive Technik wird das patientenseitige, intraoperative Risiko für Verletzungen von Nerven‑, Gefäß- sowie Organstrukturen im kleinen Becken signifikant gemindert. Zusätzlich sind die intraoperative Belastung des Patienten sowie der erwartete Blutverlust geringer.
- Hierbei besteht insbesondere für geriatrische Patienten sowie Patienten mit multiplen Komorbiditäten ein geringeres intraoperatives Risiko.
- Dies ist insbesondere bei Patienten mit multiplen Voroperationen im Bauchraum relevant, da bei hochgradigen narbigen Veränderungen ggf. das Risiko einer iatrogenen Verletzung von intraabdominellen Strukturen erhöht ist.
- Die Schraubenplanung kann hinsichtlich der technischen Realisierbarkeit aufgrund anatomischer Morphologien bereits präoperativ durchgeführt werden.
- Die präoperative Bildgebung wird durch einen intraoperativ durchgeführten 3‑D-Scan verifiziert, und die anatomischen Landmarken werden registriert. Durch die Anwendung eines Navigationssystems ist bei korrekter Anwendung eine Fehllage der Schraubenosteosynthese unwahrscheinlich.
- Durch die Nutzung des Navigationssystems lassen sich Schrauben sowohl für den supraazetabulären Bereich als auch für den vorderen und hinteren Pfeiler planen.
- Bei entsprechender Wahl des Schraubendurchmessers können die Patienten in der Regel unter Vollbelastung mobilisiert werden.

## Nachteile

- Die Indikationsstellung der minimal-invasiven und navigierten Osteosynthese des Azetabulums muss entsprechend dem Frakturmuster korrekt sein, da technisch bedingt keine aufwendigen Repositionsmanöver intraoperativ durchgeführt werden können.
- Bei Patienten mit feiner oder dysmorpher Beckenanatomie kann eine Schraubenplatzierung mit entsprechendem Durchmesser technisch schwierig bis hin zu unmöglich sein.
- Hierbei besteht gerade bei schmalem Schraubenkorridor im Azetabulumbereich ein Risiko, dass der Durchmesser der Schraube bei korrekter Drahtlage unterschätzt wird, was ggf. zu einer anschließenden intraartikulären Schraubenlage führen kann.
- Bei stark übergewichtigen Patienten ist die perkutane, navigierte Platzierung der Führungsdrähte schwierig bis unmöglich.
- Zusätzlich kann es durch unsachgemäße Handhabung der patientenseitigen Navigationsreferenz zu Abweichungen der anatomischen Strukturen mit dem auf dem Navigationssystem abgebildeten Datensatz kommen.

## Indikationen

- Die minimal-invasive, navigierte Schraubenosteosynthese kann bei einfachen oder mehrfragmentären Frakturen mit keiner oder leichter Dislokation des Azetabulums auch in Verbindung mit Frakturen des oberen und unteren Schambeinastes sowie Frakturen im Sakrum mit zusätzlicher Stabilisierung der dortiger Frakturen durchgeführt werden.

## Kontraindikationen

- Bei grob dislozierten Frakturen des Azetabulums mit Protrusion des Hüftkopfes in das kleine Becken ist die minimal-invasive und navigierte Technik kontraindiziert, da keine ausführlichen Repositionsmanöver durchgeführt werden können.
- Patienten mit angeborenen oder erworbenen anatomischen Abweichungen im Becken bieten den Schrauben ggf. keinen ausreichenden Korridor, um das Azetabulum entsprechend zu stabilisieren.

## Patientenaufklärung

- Allgemeine Operationsrisiken
- Risiko der Nachblutung und lokalen Hämatombildung bei Verletzung von Gefäßen mit Notwendigkeit der chirurgischen oder interventionell-radiologischen Gefäßversorgung
- Verletzung von im Operationsgebiet verlaufenden Nervenstrukturen mit entsprechenden motorischen und sensiblen Ausfällen
- Risiko der sekundären Dislokation der Schraubenosteosynthese mit Notwendigkeit eines Zweiteingriffes

## Operationsvorbereitungen

**Abb. 1:**
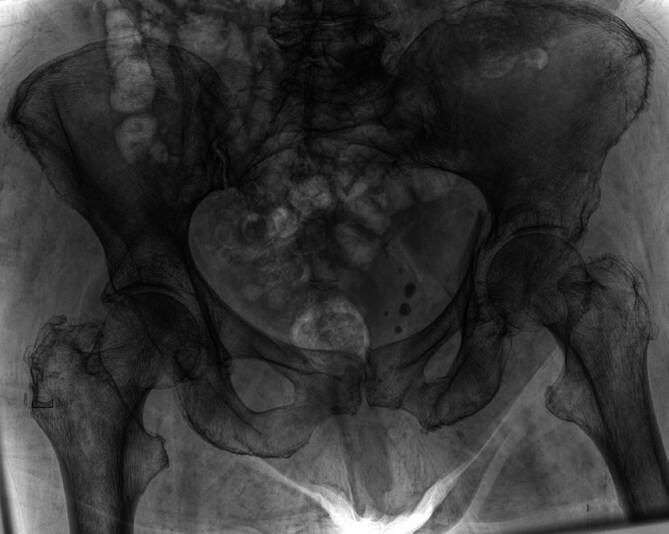
Die initiale Röntgendiagnostik kann zwar einen Hinweis auf Frakturen im Becken geben, ist jedoch für die Therapieplanung allein nicht ausreichend. Hier zeigt sich eine Azetabulumfraktur auf der linken Seite einer 89-jährigen Frau nach häuslichem Sturz, die genaue Frakturmorphologie kann jedoch nur bedingt festgestellt werden

**Abb. 2:**
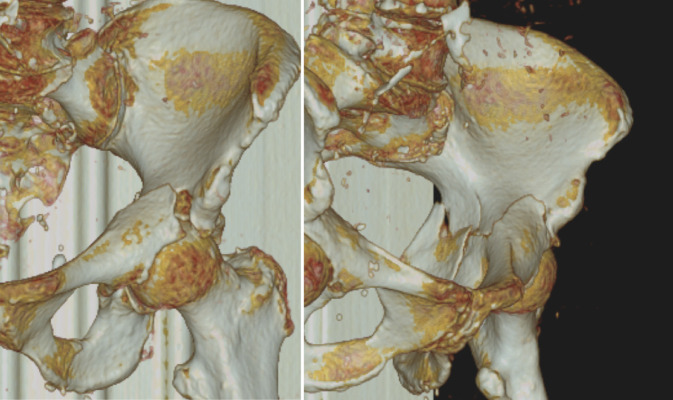
In der präoperativen CT-Diagnostik kann die genaue Frakturmorphologie festgestellt und mögliche Therapieoptionen können eruiert werden. In der Rekonstruktion stellt sich die leicht dislozierte T‑Fraktur des Azetabulums Typ 6 nach Letournel und Judet dar

- Eine Röntgendiagnostik mit Beckenübersichts‑, Ala- und Obturatoraufnahme kann initial durchgeführt werden, spielt hinsichtlich der Planung der minimal-invasiven, navigierten Versorgung jedoch nur eine untergeordnete Rolle (Abb. 1).
- Eine Computertomographie des Beckens zur genauen Darstellung der Frakturmorphologie und zur präoperativen Planung der Schraubenlage ist immer notwendig (Abb. 2).
- Elektrisches Clipping der betroffenen Beckenseite mit Glutealregion unmittelbar vor Operation

## Instrumentarium

- Für das Navigationssystem wird das entsprechende hausübliche Instrumentarium benötigt. Wir benutzen das BrainLab-Navigationssystem (Fa. BrainLab, München) in Kombination mit dem Siemens Artis Pheno-System (Fa. Siemens, Forchheim) als 3‑D-Bildwandler. Hier haben wir intraoperativ die größtmögliche Autonomie und können den C‑Bogen unabhängig des OP-Personals bedienen.
- Zur Fixierung der patientenseitigen Referenz wird eine Schanz-Schraube benötigt, welche über die Spina iliaca anterior superior im Becken fixiert wird. Die navigierte Schraubenplanung wird anschließend über den referenzierten Pointer durchgeführt.
- In der Regel nutzen wir 7,3 mm kanülierte Vollgewindeschrauben. Kurzgewindeschrauben können im Zugschraubenprinzip verwendet werden, bieten im osteoporotischen Knochen jedoch weniger Halt. Hierfür werden 2,8-mm-Führungsdrähte entsprechend der Planung vorgelegt. Mit einem 5,0 mm kanülierten Bohrer wird die laterale Kortikalis bei Bedarf aufgebohrt.

## Anästhesie und Lagerung

- Die Operation sollte, wenn möglich, in Allgemeinanästhesie durchgeführt werden. Bei multipel vorerkrankten Patienten mit hochgradig intraoperativem Risiko ist bei orientierten Patienten eine Spinalanästhesie möglich.
- Die Patienten müssen während des Eingriffs aufgrund der Referenzierung des Navigationssystems absolut ruhig liegen bleiben, weshalb andere regionale Anästhesieverfahren in der Regel nicht zielführend sind.
- Der Patient wird in der Rückenlage auf einem Carbontisch oder Tisch mit Carbonbeinteil gelagert. Es muss darauf geachtet werden, dass das Becken vollständig auf dem Carbonteil zu liegen kommt, da es sonst zu Interferenzen mit dem Röntgengerät kommt. Eine Röntgenmatte kann zum Strahlenschutz unter den Oberkörper gelegt werden, hierbei ist ebenfalls darauf zu achten, dass diese nicht das Becken überlagert.
- Die Arme werden über dem Oberkörper verschränkt fixiert, einerseits so, dass der Arm auf der betroffenen Seite nicht im OP-Zugangsgebiet zu liegen kommt, andererseits sollte der kontralaterale Arm außerhalb des Strahlengangs während des 3‑D-Scans sein. Auf eine weiche Polsterung der prominenten Stellen an Ellenbogen und Handgelenk muss geachtet werden.
- Wir hüllen den Patienten ab Höhe des Beckens über die gesamte Länge der Beine zirkulär in sterile Abdecktücher ein. Dies verhindert ein Anheben der sterilen Abdeckung durch den bodengebundenen C‑Bogen beim 3‑D-Scan.
- Die Kamera des Navigationssystems muss entsprechend der Infrastruktur im OP auf die Seite der Fraktur bewegt werden, sodass während der OP keine Interferenzen zwischen Kamera und Instrumentarium durch beispielsweise Abdecktücher entstehen.

## Operationstechnik

Abb. 3, 4, 5, 6, 7, 8, 9, 10, 11 und 12

**Abb. 3:**
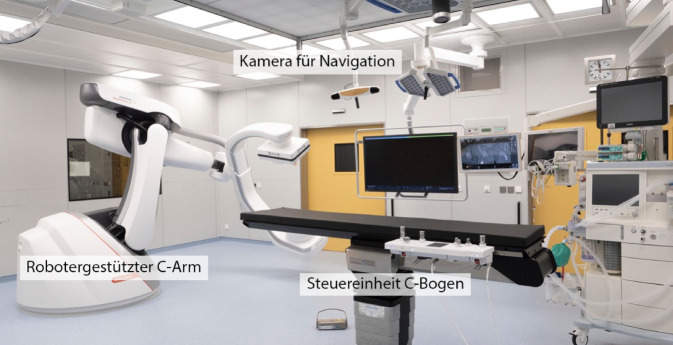
Aufbau des Operationssaals mit dem robotergestützten, *bodengebundenen C‑Bogen*, *Navigationssystem* sowie dem OP-Tisch mit *Steuereinheit des C‑Bogens*

**Abb. 4:**
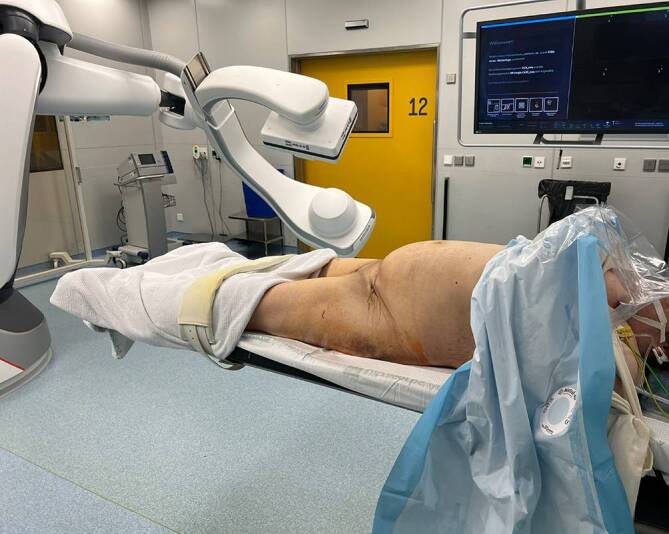
Lagerung der Patientin in Rückanlage. Die Beine werden zirkulär eingewickelt, um eine Interferenz mit der robotergestützten, bildgebenden Einheit zu vermeiden

**Abb. 5:**
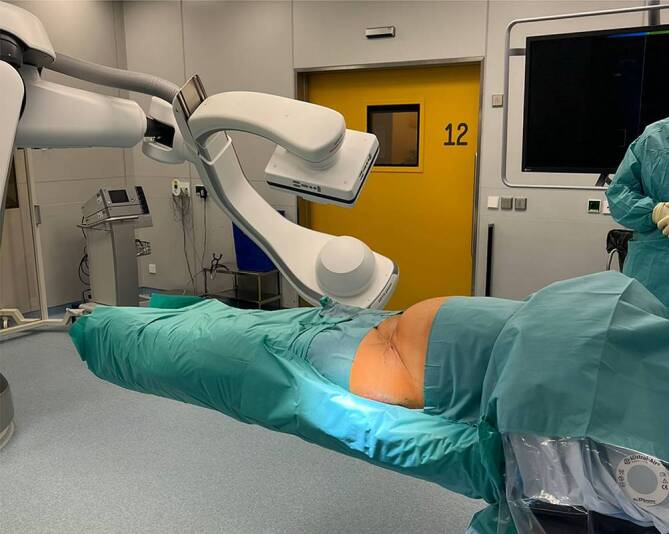
Anschließend wird die Patientin nach der Vorbereitung sowie dem Reinigen des Operationsgebiets in den sterilen Tüchern „mumifiziert“. Die sterile Abdeckung wird zirkulär um die Patientin gewickelt. Dabei wird das sterile Doppelklebetuch von den Operateuren unter dem Operationstisch durchgezogen und auf der Oberseite festgeklebt. Somit kann der robotergestützte C‑Arm um die Patientin rotieren, ohne die sterilen Tücher dabei anzuheben

**Abb. 6:**
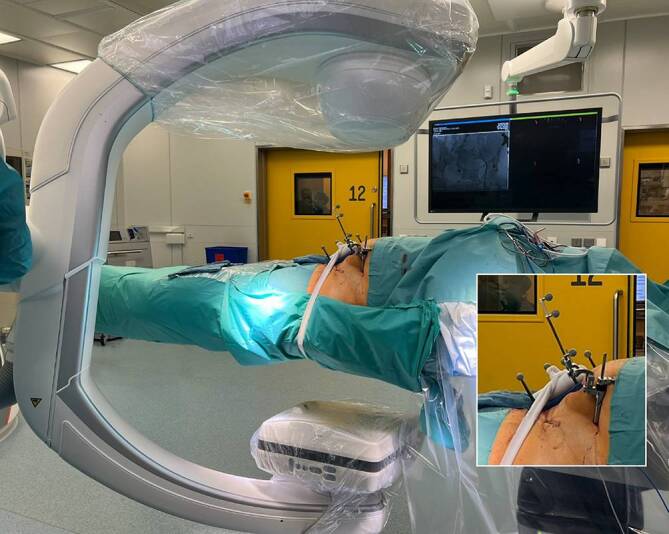
Nach Anbringen der patientenseitigen Referenz in der linken Spina iliaca anterior superior über eine Schanz-Schraube und Vorbereitung des Navigationssystems (s. Ausschnitt) wird der 3‑D-Scan mit dem C‑Bogen durchgeführt. Dieser befindet sich in der vorliegenden Abbildung bereits in Startposition und rotiert zur Durchführung 270° um den Patienten. Aus Strahlenschutzgründen verlässt das OP-Personal den Operationssaal während des Scans

**Abb. 7:**
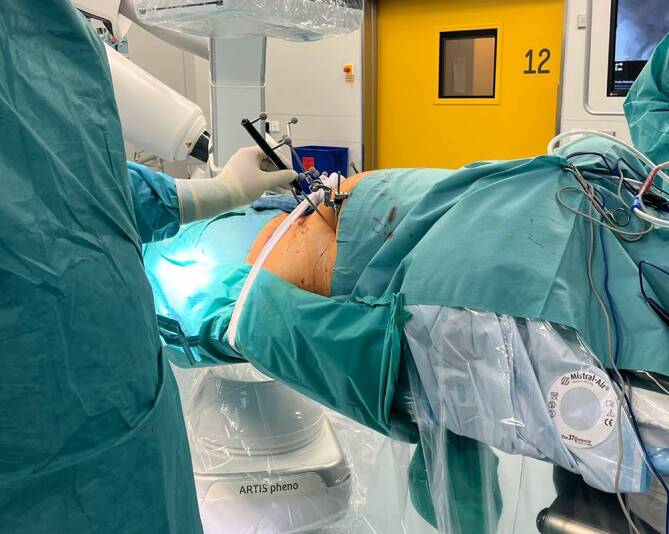
Nun wird zunächst der durchgeführte 3‑D-Scan referenziert, und anschließend werden mit dem Pointerinstrumentarium über das Navigationssystem die möglichen Schrauben visuell geplant und platziert. Hier sollte insbesondere die Anatomie des Patienten beachtet werden. Gerade bei Osteosynthesen der hinteren Azetabulumwand muss auf den Verlauf des N. ischiadicus geachtet werden

**Abb. 8:**
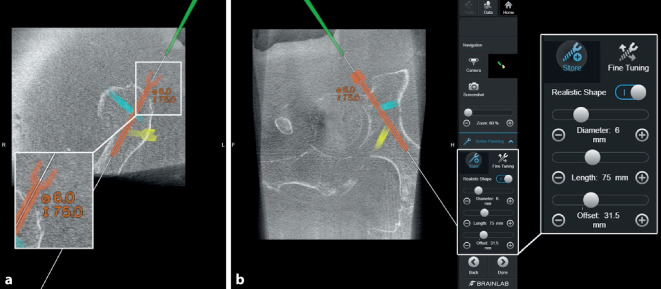
Über das Interface des Navigationssystems werden mithilfe des Pointerinstrumentariums mögliche Schraubenpositionen eruiert und geplant. Hierbei können bereits die Schraubenlänge und Durchmesser kontrolliert (**a**) und über das Menü bei Bedarf angepasst (**b**) und eventuelle Schraubenkorridore so geprüft werden

**Abb. 9:**
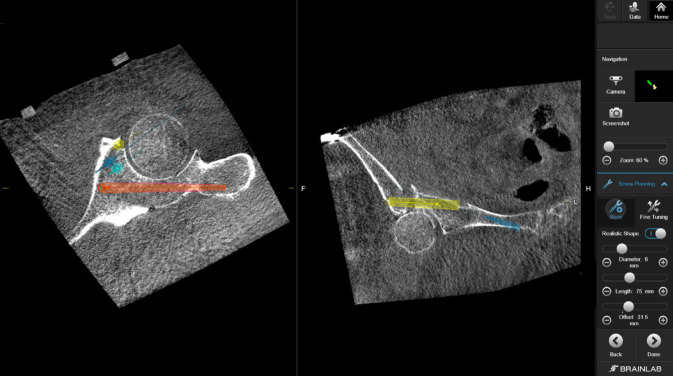
Die Planung der Schrauben kann dann nochmals manuell über den Touchscreen feinjustiert werden, um eventuelle intraartikuläre Fehllagen zu vermeiden

**Abb. 10:**
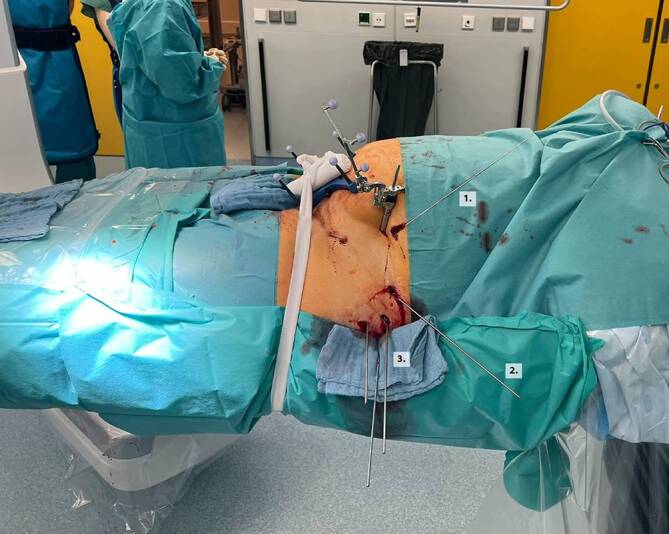
Über ein navigiertes Zielinstrumentarium können nun über Stichinzisionen die Führungsdrähte entlang der geplanten Schraubenosteosynthesen gebohrt werden. Für die Platzierung des Führungsdrahtes in den hinteren Pfeiler *(1.)* ist ein minimal-invasiver Zugang zum ersten Letournel-Fenster notwendig. Der Draht für den vorderen Pfeiler *(2.)* kann nur bei ausreichendem anatomischem Korridor gesetzt werden, um eine intraartikuläre Lage der Schraube zu vermeiden. In der Regel versuchen wir mindestens 2 supraazetabuläre Schrauben zu platzieren, um eine höhere Stabilität zu erreichen. In diesem Fall wurden 3 Drähte vorgelegt *(3.)*

**Abb. 11:**
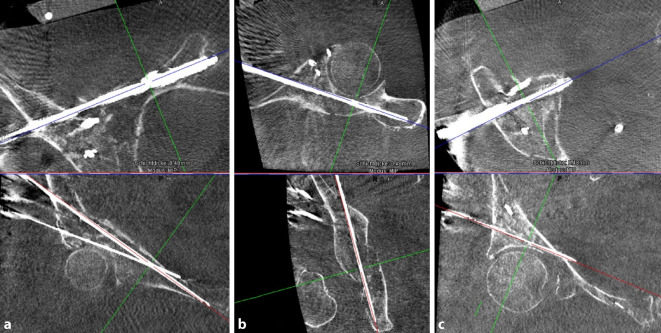
Nach navigierter Implantation der Drähte erfolgt eine erneute Kontrolle über den 3‑D-Scan, um mögliche Fehllagen auszuschließen und ggf. korrigieren zu können. Die Bildebenen können vom Operateur steril im OP eingestellt werden. Abgebildet zeigen sich die Führungsdrähte für die vordere Pfeilerschraube (**a**), für die hintere Pfeilerschraube (**b**) sowie für die supraazetabuläre Schraube (**c**) jeweils in 2 Ebenen (*oben* und *unten*)

**Abb. 12:**
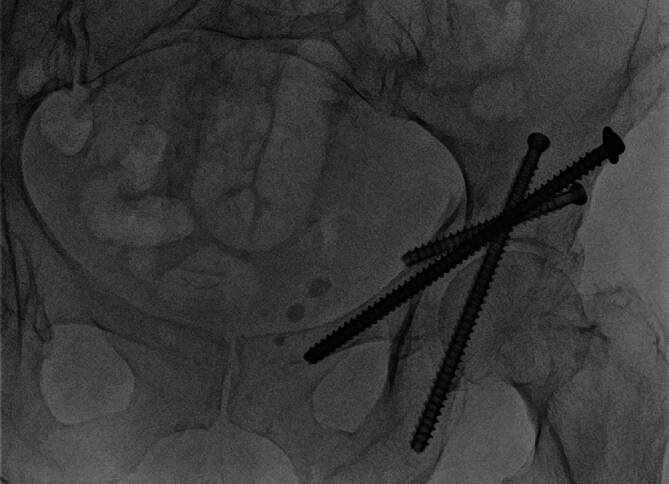
Das intraoperative Bild nach Schraubenimplantation zeigt die korrekte Schraubenlage bei Fixierung aller Fragmente. Eine intraartikuläre Lage der Schrauben kann im Röntgenbild nicht suffizient ausgeschlossen werden, weshalb dies bereits im intraoperativen 3‑D-Scan durchgeführt wird

## Postoperative Behandlung

**Abb. 13:**
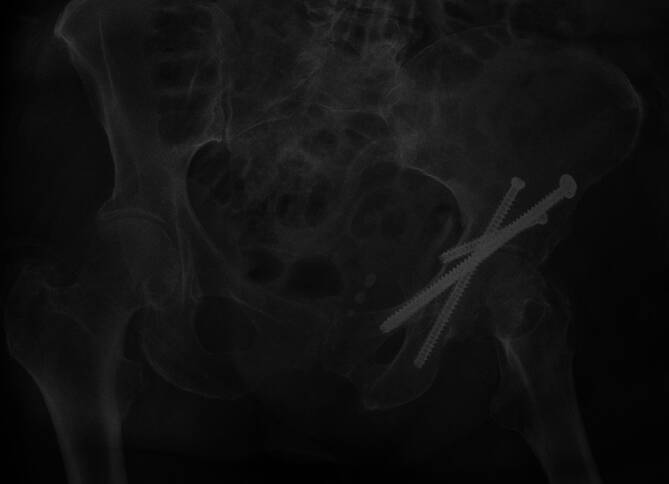
Die Kontrolle nach 6 Wochen dokumentiert die korrekte Lage der Schrauben ohne eine sekundäre Dislokation. Die Patientin kann sich bereits selbstständig wieder am Rollator unter Vollbelastung des linken Beines mobilisieren

- Das Fadenmaterial kann bei gesicherter Wundheilung zeitgerecht entfernt werden. Ein einfacher Pflasterverband ist bis dahin ausreichend.
- Bereits am ersten postoperativen Tag erfolgt die physiotherapeutische Beübung in der Regel mit schmerzadaptierter Vollbelastung am hohen Gehwagen.
- Bei sicherer Mobilisierung am hohen Gehwagen kann anschließend entsprechend den Schmerzen der Patienten auf Unterarmgehstützen gewechselt werden.
- Wir empfehlen die radiologischen Kontrollen nach 6 Wochen (Abb. 13), 3 Monaten und nach 12 Monaten.
- Eine Metallentfernung ist in der Regel nicht notwendig.

## Fehler, Gefahren, Komplikationen

- Eine sekundäre Dislokation der Schraubenosteosynthesen bzw. der Frakturfragmente bedarf in der Regel einer Revisionsoperation ggf. mit offener Reposition und Plattenosteosynthese.
- Intraoperative Verletzungen von Gefäßen und Nerven lassen sich durch die minimal-invasive Technik nur bedingt adressieren. Hier ist die offene Revision sinnvoll, bei Gefäßverletzungen ggf. auch sekundär über die radiologische Intervention.

## Ergebnisse

Im Zeitraum von Januar 2015 bis Dezember 2023 wurden 101 Patienten mit minimal oder nicht dislozierten Azetabulumfrakturen mit Unterstützung des Navigationssystems sowie der robotergestützten bildgebenden Einheit operativ mittels minimal-invasiver Schraubenosteosynthese versorgt. Davon waren 59 Patienten weiblich und 42 Patienten männlich. Das Durchschnittsalter lag bei 71,1 Jahren (17 bis 98 Jahre). Die Patienten wurden nach stationärer Aufnahme innerhalb von durchschnittlich 3,1 Tagen operativ versorgt und verblieben im Schnitt 9,1 Tage (9 bis 26 Tage) in stationärer Betreuung. Der Charité Mobility Index (CHARMI) der Patienten lag im Durchschnitt vor Trauma bei 9,2. Die Patienten wurden alle täglich unter physiotherapeutischer Anleitung mobilisiert. Vor Entlassung konnte ein durchschnittlicher CHARMI von 6,7 gemessen werden; 79 % aller Patienten mit Follow-up konnten wieder in ihr gewohntes Umfeld entlassen werden. Für 21 % der Patienten war eine Entlassung in die Häuslichkeit aufgrund fehlender Unterstützung nicht möglich, und sie benötigten eine kurzzeitige oder dauerhafte Pflege in einem Heim.

Von 101 Patienten konnten 86 Patienten nach 6 Wochen klinisch und radiologisch nachkontrolliert werden. Für diese Patienten betrug das durchschnittliche Follow-up 49,7 Wochen (6 bis 79 Wochen). Bei einer Patientin zeigte sich im Verlauf eine sekundäre Dislokation einer Schraubenosteosynthese, weshalb eine Revisionsoperation mit Neuplatzierung notwendig war. Bei einer weiteren Patientin zeigte sich eine sekundäre Dislokation einer Schraubenosteosynthese mit intraartikulärer Lage, weshalb nach 6 Wochen postoperativ die Indikation zur Hüftgelenkendoprothese gestellt wurde.

In der Literatur werden gerade die T‑Frakturen im Azetabulum zwar als selten, jedoch besonders Komplikationsreich beschrieben. Die offene Reposition und Plattenosteosynthese gehen mit einem hohen peri- und postoperativen Risiko einher [3, 6]. Als Alternative wird auch hier die minimal-invasive Schraubenosteosynthese genannt [4]. Die Literatur beschreibt in einigen biomechanischen Untersuchungen, dass es keinen signifikanten Unterschied der Stabilität einer Plattenosteosynthese verglichen mit minimal-invasiven Schraubenosteosynthesen hinsichtlich der Dislokationsrate unter Belastung auch bei T‑Frakturen gibt. Becker et al. konnten in ihrer biomechanischen Studie keine signifikanten Unterschiede zwischen der Schrauben‑, Plattenosteosynthese auch in Kombination mittels internen Fixateurs feststellen [1]. Die Grenzen der minimal-invasiven Technik waren in unserer Patientenklientel insbesondere die dislozierten Frakturen.

Zusammenfassend bietet die minimal-invasive Versorgung von nicht oder minimal dislozierten Azetabulumfrakturen unter Zuhilfenahme eines robotergestützten 3‑D-C-Bogens sowie Navigationssystems eine moderne, valide Alternative gerade für die geriatrische Patientenklientel mit geringem peri- und postoperativem Risiko.
